# Crystal structure of chlorido­(dimethyl sulfoxide-κ*S*)bis­[4-(pyridin-2-yl)benzaldehyde-κ^3^ *C* ^2^,*N*]iridium(III) aceto­nitrile monosolvate

**DOI:** 10.1107/S2056989017010945

**Published:** 2017-08-01

**Authors:** Andrew J. Peloquin, Madelyn B. Smith, Gary J. Balaich, Scott T. Iacono

**Affiliations:** a Department of Chemistry & Chemistry Research Center, United States Air Force Academy, Colorado Springs, CO 80840, USA

**Keywords:** crystal structure, iridium, phenyl­pyridine derivative

## Abstract

The title compound, [IrCl(C_12_H_8_NO)_2_{(CH_3_)_2_SO}]·H_3_CCN or [IrCl(fppy)_2_(DMSO)]·H_3_CCN [where fppy is 4-(pyridin-2-yl)benzaldehyde and DMSO is dimethyl sulfoxide], is a mononuclear iridium(III) complex including two fppy ligands, a sulfur-coordinating DMSO ligand, and one terminal chloride ligand that define a distorted octa­hedral coordination sphere. The complex crystallizes from 1:1 DMSO–aceto­nitrile as an aceto­nitrile solvate. In the crystal, weak C—H⋯O and C—H⋯N hydrogen-bonding inter­actions between adjacent complexes and between the aceto­nitrile solvent and the complex consolidate the packing.

## Abstract 2

The title iridium(III) complex crystallizes as a aceto­nitrile monosolvate and has the Ir^III^ atom in a distorted octa­hedral coordination within a C_2_N_2_ClS coordination set.

## Chemical context   

The development of iridium complexes with three *ortho* metallating ligands has drawn great inter­est due to their potential application in light-emitting devices (Henwood & Zysman-Coman, 2017 ▸). Many such complexes have been synthesized, often utilizing phenyl­pyridine-based di­chlorido-bridged di-iridium complexes as starting materials. In an attempt to synthesize such a compound with 4-(pyridin-2-yl)benzaldehyde (fppy) as a ligand, *viz*. di-*μ*-chlorido-bis{bis­[4-(pyridin-2-yl)benzaldehyde-*κ* ^2^ *C* ^2^,*N′*]iridium(III) (Bet­­tington *et al.*, 2004 ▸), spectroscopic results indicated a product with reduced symmetry compared to the expected *C_i_* symmetry of the known complex. Single-crystal X-ray analysis was used to elucidate the structure of the title compound.
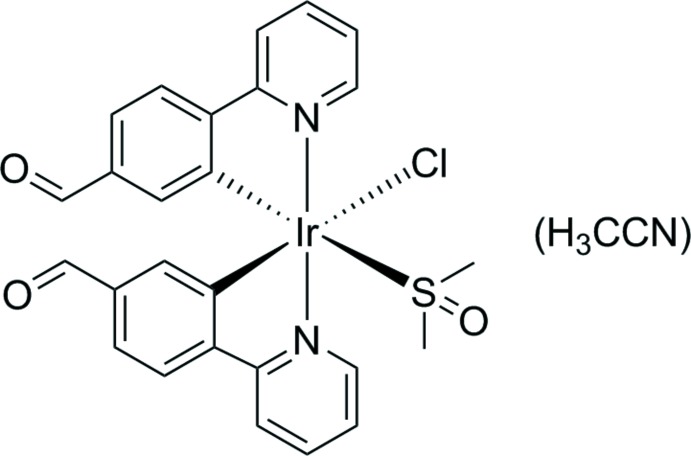


## Structural commentary   

**Figure 1:**
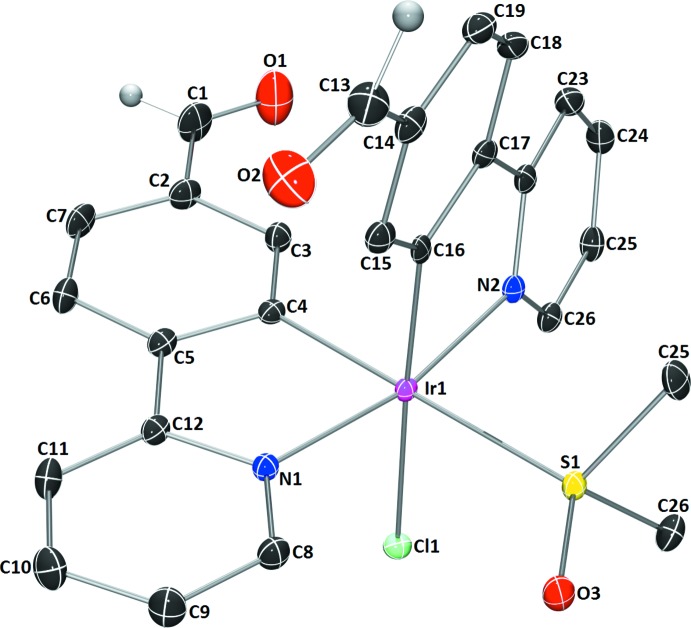
The mol­ecular structure of chlorido­(dimethyl sulfoxide-κ*S*)bis­[4-(pyridin-2-yl)benzaldehyde-κ^2^ *C* ^2^,*N*′]iridium(III) aceto­nitrile monosolvate. Displacement ellipsoids are shown at the 50% probability level. Only aldehyde H atoms are shown and the aceto­nitrile solvent mol­ecule has been omitted for clarity.

The title compound (Fig. 1 ▸) crystallizes in the triclinic space group *P* 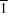 with one mol­ecule per asymmetric unit. The Ir^III^ atom has a distorted octa­hedral coordination sphere defined by the S atom of the dimethyl sulfoxide (DMSO) ligand, a chlorine ligand and C and N atoms of two fppy ligands. The S and Cl atoms occupy equatorial positions, *trans* to the fppy C atoms, and the fppy N atoms occupy the axial positions. The least-squares planes of each fppy ligand indicate a nearly coplanar arrangement of the pyridine and phenyl rings with small deviations of 2.42 (9)° (fppy ligand N1/C2–C12 with C4 *trans* to S1) and 14.71 (9)° (fppy ligand N2/C14–C24 with C16 *trans* to Cl1). The Ir—S bond length [2.3810 (5) Å] is longer than the average distance [2.27 (1) Å] that was reported for this coordination mode (Calligaris, 2004 ▸). The S—O distance [1.4903 (13) Å] is only slightly longer than the previously reported average [1.473 (4) Å]. This S—O distance shows negligible contraction from the average reported S—O bond length [1.492 (1) Å] in non-coordinating sulfoxide mol­ecules (Calligaris, 2004 ▸).

## Supra­molecular features   

**Figure 2:**
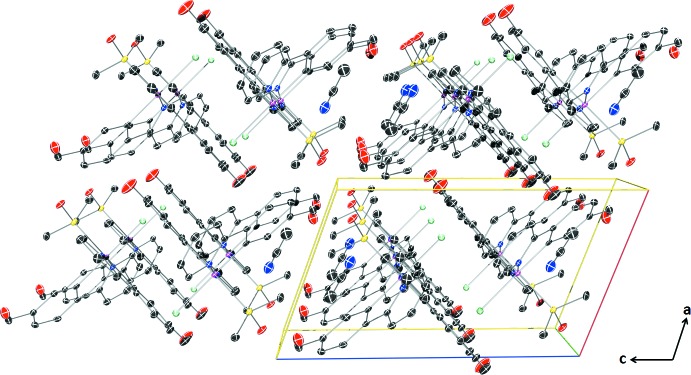
The crystal packing of the title complex, viewed along the *b* axis. Displacement ellipsoids are shown at the 50% probability level. H atoms have been omitted for clarity.

The aceto­nitrile solvate mol­ecules fill voids that are visible along the *b* axis view direction (Fig. 2 ▸). Alignment of the aceto­nitrile mol­ecules is caused by weak inter­molecular contacts between aceto­nitrile H atoms and adjacent fppy aldehyde carbonyl O atoms (C28—H28⋯O1) in addition to weak inter­actions between aceto­nitrile N atoms and adjacent DMSO H atoms (C25—H25*B*⋯N3). There are also C—H⋯O inter­actions between adjacent complexes, involving aromatic H atoms of one of the fppy ligands and methyl groups of the DMSO ligand with the sulfoxide O atom. Numerical details of all these inter­actions are collated in Table 1 ▸.

## Database survey   

A search of the Cambridge Structural Database (CSD, V5.38, update February 2017; Groom *et al.*, 2016 ▸) for related structures revealed that the di-*μ*-chlorido-bis­{bis­[4-(pyridin-2-yl)benzaldehyde-*κ* ^2^ *C* ^2^,*N′*]iridium(III)} complex from which the title complex was derived, has been reported as a di­chloro­methane sesquisolvate (Bettington *et al.*, 2004 ▸).

## Synthesis and crystallization   

The parent compound, di-*μ*-chlorido-bis­{bis­[4-(pyridin-2-yl)benzaldehyde-*κ* ^2^ *C* ^2^,*N′*]iridium(III), was synthesized utilizing a previously reported procedure (Bettington *et al.*, 2004 ▸).

For the synthesis of the title compound, di-*μ*-chlorido-bis­{bis­[4-(pyridin-2-yl)benzaldehyde-k^2^ *C* ^2^,*N′*]iridium(III) (0.101 g, 0.077 mmol) was dissolved in DMSO (2 ml) with gentle heating over 5 min. After cooling to room temperature, aceto­nitrile (2 ml) was added. After 24 h, the resulting solid was collected by vacuum filtration to afford the title compound as an orange crystalline solid (0.043 g, 41.7%). Spectroscopic data: ^1^H NMR (500 MHz, DMSO-*d*_6_): δ 9.83 (*d*, 1H, *J* = 8.0 Hz), 9.61 (*s*, 1H), 9.55–9.53 (*m*, 2H), 8.42 (*d*, 1H, *J* = 8.0 Hz), 8.34 (*d*, 1H, *J* = 8.0 Hz), 8.20 (*t*, 1H, *J* = 7.5 Hz), 8.11 (*t*, 1H, *J* = 7.5 Hz), 8.01 (*d*, 1H, *J* = 7.5 Hz), 7.96 (*d*, 1H, *J* = 8.0 Hz), 7.69 (*t*, 1H, *J* = 6.5 Hz), 7.60 (*t*, 1H, *J* = 6.5 Hz), 7.40 (*d*, 1H, *J* = 8.0 Hz), 7.36 (*d*, 1H, *J* = 8.0 Hz), 6.71 (*s*, 1H), 6.10 (*s*, 1H) and ^13^C NMR (500 MHz, DMSO-*d*_6_): δ 193.6, 193.5, 166.1, 165.7, 153.1, 152.1, 151.7, 150.5, 149.9, 145.5, 140.4, 139.4, 136.6, 135.9, 131.2, 129.4, 126.4, 125.9, 125.7, 125.5, 125.2, 124.8, 122.4, 121.8, 72.3, 66.0, 60.8, 40.5, 40.3, 40.1, 40.0, 39.8, 39.6, 39.5, 15.7.

## Refinement   

Crystal data as well as data collection and structure refinement details are summarized in Table 2 ▸. The aldehyde hydrogen atoms were found in a difference-Fourier map and were refined freely. The remaining hydrogen atoms were included in calculated positions and refined with a riding model: C—H = 0.95-0.98 Å with *U*_iso_(H) = 1.5 *U*_eq_(C-meth­yl) and 1.2 *U*_eq_(C) for other H atoms.

## Supplementary Material

Crystal structure: contains datablock(s) global, I. DOI: 10.1107/S2056989017010945/wm5400sup1.cif

Structure factors: contains datablock(s) I. DOI: 10.1107/S2056989017010945/wm5400Isup2.hkl

CCDC reference: 1564599

Additional supporting information:  crystallographic information; 3D view; checkCIF report

## Figures and Tables

**Table 1:** Hydrogen-bond geometry (Å, °)

*D*—H⋯*A*	*D*—H	H⋯*A*	*D*⋯*A*	*D*—H⋯*A*
C19—H19⋯O3^i^	0.93	2.48	3.179 (3)	132
C25—H25*B*⋯N3^ii^	0.96	2.53	3.403 (3)	150
C26—H26*A*⋯O3^iii^	0.96	2.48	3.406 (2)	161
C28—H28*C*⋯O1^iv^	0.96	2.54	3.490 (3)	173

Symmetry codes: (i) 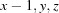; (ii) 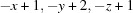; (iii) 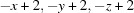; (iv) 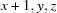.

**Table 2:** Experimental details

Crystal data	
Chemical formula	[IrCl(C_12_H_8_NO)_2_(C_2_H_6_OS)]·C_2_H_3_N
*M*_r_	711.22
Crystal system, space group	Triclinic, *P* 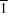
Temperature (K)	100
*a*, *b*, *c* (Å)	8.7837 (12), 12.0910 (16), 14.0097 (19)
α, β, γ (°)	97.5367 (15), 105.1501 (14), 109.3176 (14)
*V* (Å^3^)	1316.1 (3)
*Z*	2
Radiation type	Mo *K*α
μ (mm^−1^)	5.29
Crystal size (mm)	0.32 × 0.24 × 0.17

Data collection	
Diffractometer	Bruker SMART APEX CCD
Absorption correction	Multi-scan (*SADABS*; Krause *et al.*, 2015 ▸)
*T*_min_, *T*_max_	0.26, 0.47
No. of measured, independent and observed [*I* > 2σ(*I*)] reflections	28976, 6983, 6828
*R*_int_	0.026
(sin θ/λ)_max_ (Å^−1^)	0.685

Refinement	
*R*[*F* ^2^ > 2σ(*F* ^2^)], *wR*(*F* ^2^), *S*	0.015, 0.037, 1.68
No. of reflections	6983
No. of parameters	345
H-atom treatment	H atoms treated by a mixture of independent and constrained refinement
Δρ_max_, Δρ_min_ (e Å^−3^)	1.47, −0.65

Computer programs: *APEX3* and *SAINT* (Bruker, 2017 ▸), *SHELXS* (Sheldrick, 2008 ▸), *SHELXL2016* (Sheldrick, 2015 ▸), *ORTEP-3 for Windows* (Farrugia, 2012 ▸) and *Mercury* (Macrae *et al.*, 2008 ▸).

## supplementary crystallographic information

### Crystal data

**Table:**

[IrCl(C_12_H_8_NO)_2_(C_2_H_6_OS)]·C_2_H_3_N	*Z* = 2
*M**_r_* = 711.22	*F*(000) = 696
Triclinic, *P*1	*D*_x_ = 1.795 Mg m^−^^3^
*a* = 8.7837 (12) Å	Mo *K*α radiation, λ = 0.71073 Å
*b* = 12.0910 (16) Å	Cell parameters from 9979 reflections
*c* = 14.0097 (19) Å	θ = 2.6–30.0°
α = 97.5367 (15)°	µ = 5.29 mm^−^^1^
β = 105.1501 (14)°	*T* = 100 K
γ = 109.3176 (14)°	Rectangular prism, orange
*V* = 1316.1 (3) Å^3^	0.32 × 0.24 × 0.17 mm

### Data collection

**Table:**

Bruker SMART APEX CCD diffractometer	6983 independent reflections
Radiation source: fine focus sealed tube	6828 reflections with *I* > 2σ(*I*)
Graphite monochromator	*R*_int_ = 0.026
Detector resolution: 8.3333 pixels mm^-1^	θ_max_ = 29.1°, θ_min_ = 1.8°
ω Scans scans	*h* = −11→11
Absorption correction: multi-scan (*SADABS*; Krause *et al.*, 2015)	*k* = −16→16
*T*_min_ = 0.26, *T*_max_ = 0.47	*l* = −19→19
28976 measured reflections	

### Refinement

**Table:**

Refinement on *F*^2^	Primary atom site location: structure-invariant direct methods
Least-squares matrix: full	Secondary atom site location: difference Fourier map
*R*[*F*^2^ > 2σ(*F*^2^)] = 0.015	Hydrogen site location: mixed
*wR*(*F*^2^) = 0.037	H atoms treated by a mixture of independent and constrained refinement
*S* = 1.68	*w* = 1/[σ^2^(*F*_o_^2^)] where *P* = (*F*_o_^2^ + 2*F*_c_^2^)/3
6983 reflections	(Δ/σ)_max_ = 0.003
345 parameters	Δρ_max_ = 1.47 e Å^−^^3^
0 restraints	Δρ_min_ = −0.65 e Å^−^^3^

### Special details

**Table:**

Geometry. All esds (except the esd in the dihedral angle between two l.s. planes) are estimated using the full covariance matrix. The cell esds are taken into account individually in the estimation of esds in distances, angles and torsion angles; correlations between esds in cell parameters are only used when they are defined by crystal symmetry. An approximate (isotropic) treatment of cell esds is used for estimating esds involving l.s. planes.

### Fractional atomic coordinates and isotropic or equivalent isotropic displacement parameters (Å^2^)

**Table:**

	*x*	*y*	*z*	*U*_iso_*/*U*_eq_	
C1	−0.0050 (3)	0.6613 (2)	0.33167 (17)	0.0243 (5)	
H1	−0.088 (3)	0.596 (2)	0.278 (2)	0.042 (8)*	
C2	0.1092 (2)	0.62505 (18)	0.40746 (15)	0.0157 (4)	
H2	0.077 (3)	0.594 (2)	0.9857 (18)	0.028 (6)*	
C3	0.2305 (2)	0.71022 (17)	0.49507 (14)	0.0134 (4)	
H3	0.239758	0.790067	0.505177	0.016*	
C4	0.3376 (2)	0.67614 (16)	0.56730 (14)	0.0100 (3)	
C5	0.3222 (2)	0.55552 (16)	0.54791 (14)	0.0116 (4)	
C6	0.2015 (2)	0.46989 (17)	0.46076 (14)	0.0151 (4)	
H6	0.193066	0.390228	0.449892	0.018*	
C7	0.0941 (2)	0.50534 (17)	0.39041 (15)	0.0164 (4)	
H7	0.012541	0.449242	0.332285	0.02*	
C8	0.6532 (2)	0.60005 (17)	0.78483 (14)	0.0134 (4)	
H8	0.724573	0.664417	0.840195	0.016*	
C9	0.6625 (2)	0.48877 (17)	0.78530 (15)	0.0167 (4)	
H9	0.737233	0.47808	0.840651	0.02*	
C10	0.5597 (3)	0.39315 (17)	0.70259 (15)	0.0175 (4)	
H10	0.566006	0.317648	0.70063	0.021*	
C11	0.4471 (2)	0.41157 (16)	0.62266 (15)	0.0153 (4)	
H11	0.37664	0.348031	0.566469	0.018*	
C12	0.4389 (2)	0.52483 (16)	0.62615 (14)	0.0112 (3)	
C13	0.1550 (3)	0.57976 (18)	0.94959 (15)	0.0176 (4)	
C14	0.1860 (2)	0.66267 (16)	0.88152 (14)	0.0128 (4)	
C15	0.3053 (2)	0.66650 (16)	0.83096 (14)	0.0122 (4)	
H15	0.363082	0.614479	0.838132	0.015*	
C16	0.3375 (2)	0.74788 (16)	0.77005 (13)	0.0098 (3)	
C17	0.2417 (2)	0.82202 (16)	0.75755 (14)	0.0108 (3)	
C18	0.1194 (2)	0.81540 (17)	0.80579 (14)	0.0133 (4)	
H18	0.055266	0.863072	0.795451	0.016*	
C19	0.0948 (2)	0.73674 (16)	0.86940 (14)	0.0143 (4)	
H19	0.016858	0.733882	0.903893	0.017*	
C20	0.5027 (2)	1.00524 (16)	0.62983 (14)	0.0120 (4)	
H20	0.605771	1.014028	0.61919	0.014*	
C21	0.4188 (2)	1.07846 (16)	0.59468 (14)	0.0141 (4)	
H21	0.464917	1.135452	0.560855	0.017*	
C22	0.2647 (2)	1.06543 (17)	0.61075 (14)	0.0157 (4)	
H22	0.204513	1.111646	0.585708	0.019*	
C23	0.2021 (2)	0.98295 (17)	0.66440 (14)	0.0145 (4)	
H23	0.100937	0.97487	0.67742	0.017*	
C24	0.2915 (2)	0.91196 (16)	0.69888 (14)	0.0111 (3)	
C25	0.6344 (2)	0.96111 (17)	0.94371 (14)	0.0157 (4)	
H25A	0.559874	0.894382	0.96093	0.024*	
H25B	0.570208	1.004318	0.911162	0.024*	
H25C	0.722715	1.014181	1.004546	0.024*	
C26	0.8560 (2)	1.04751 (17)	0.84339 (15)	0.0164 (4)	
H26A	0.94507	1.092567	0.906542	0.025*	
H26B	0.786226	1.092503	0.823195	0.025*	
H26C	0.905534	1.033313	0.791846	0.025*	
C27	0.5454 (3)	0.76301 (18)	0.12214 (16)	0.0216 (4)	
C28	0.6709 (3)	0.7132 (2)	0.1106 (2)	0.0364 (6)	
H28A	0.723039	0.747741	0.063586	0.055*	
H28B	0.615895	0.62727	0.085237	0.055*	
H28C	0.756838	0.731849	0.175519	0.055*	
Cl1	0.74293 (5)	0.83320 (4)	0.62149 (3)	0.01155 (8)	
Ir1	0.51788 (2)	0.78209 (2)	0.70247 (2)	0.00795 (2)	
N1	0.54447 (18)	0.61957 (13)	0.70713 (11)	0.0097 (3)	
N2	0.43895 (18)	0.92185 (13)	0.67888 (11)	0.0097 (3)	
N3	0.4464 (3)	0.80168 (17)	0.13125 (15)	0.0286 (4)	
O1	−0.0021 (2)	0.76266 (15)	0.33564 (13)	0.0390 (4)	
O2	0.21023 (19)	0.50165 (13)	0.95953 (12)	0.0254 (3)	
O3	0.84599 (17)	0.85806 (12)	0.92054 (10)	0.0171 (3)	
S1	0.72772 (6)	0.90632 (4)	0.85936 (3)	0.01024 (8)	

### Atomic displacement parameters (Å^2^)

**Table:**

	*U*^11^	*U*^22^	*U*^33^	*U*^12^	*U*^13^	*U*^23^
C1	0.0214 (11)	0.0248 (12)	0.0157 (11)	0.0071 (9)	−0.0064 (9)	−0.0016 (9)
C2	0.0133 (9)	0.0191 (10)	0.0105 (9)	0.0047 (8)	0.0002 (7)	0.0014 (8)
C3	0.0139 (9)	0.0122 (9)	0.0120 (9)	0.0044 (7)	0.0025 (7)	0.0009 (7)
C4	0.0090 (8)	0.0121 (8)	0.0086 (8)	0.0029 (7)	0.0042 (7)	0.0018 (7)
C5	0.0110 (8)	0.0134 (9)	0.0089 (9)	0.0030 (7)	0.0032 (7)	0.0018 (7)
C6	0.0161 (9)	0.0110 (9)	0.0128 (9)	0.0017 (7)	0.0024 (8)	−0.0008 (7)
C7	0.0147 (9)	0.0165 (10)	0.0096 (9)	0.0012 (8)	−0.0010 (7)	−0.0022 (7)
C8	0.0123 (9)	0.0137 (9)	0.0116 (9)	0.0046 (7)	0.0007 (7)	0.0018 (7)
C9	0.0192 (10)	0.0158 (10)	0.0149 (10)	0.0082 (8)	0.0020 (8)	0.0056 (8)
C10	0.0227 (10)	0.0124 (9)	0.0184 (10)	0.0075 (8)	0.0069 (8)	0.0050 (8)
C11	0.0191 (10)	0.0101 (9)	0.0127 (9)	0.0024 (7)	0.0040 (8)	0.0002 (7)
C12	0.0111 (8)	0.0125 (9)	0.0085 (9)	0.0029 (7)	0.0033 (7)	0.0011 (7)
C13	0.0199 (10)	0.0180 (10)	0.0151 (10)	0.0048 (8)	0.0089 (8)	0.0043 (8)
C14	0.0124 (9)	0.0130 (9)	0.0084 (9)	0.0012 (7)	0.0015 (7)	0.0007 (7)
C15	0.0112 (8)	0.0124 (9)	0.0098 (9)	0.0033 (7)	0.0007 (7)	0.0008 (7)
C16	0.0094 (8)	0.0086 (8)	0.0070 (8)	0.0032 (7)	−0.0019 (7)	−0.0025 (7)
C17	0.0088 (8)	0.0112 (8)	0.0080 (8)	0.0019 (7)	−0.0005 (7)	−0.0008 (7)
C18	0.0092 (8)	0.0154 (9)	0.0131 (9)	0.0052 (7)	0.0010 (7)	0.0000 (7)
C19	0.0106 (9)	0.0160 (9)	0.0133 (9)	0.0020 (7)	0.0044 (7)	0.0003 (7)
C20	0.0127 (9)	0.0114 (9)	0.0089 (9)	0.0024 (7)	0.0021 (7)	0.0011 (7)
C21	0.0194 (9)	0.0103 (9)	0.0099 (9)	0.0044 (7)	0.0023 (7)	0.0023 (7)
C22	0.0184 (10)	0.0141 (9)	0.0132 (9)	0.0093 (8)	−0.0008 (8)	0.0024 (8)
C23	0.0122 (9)	0.0161 (9)	0.0135 (9)	0.0063 (7)	0.0016 (7)	0.0009 (8)
C24	0.0111 (8)	0.0103 (8)	0.0083 (9)	0.0031 (7)	0.0005 (7)	−0.0014 (7)
C25	0.0198 (10)	0.0157 (9)	0.0099 (9)	0.0069 (8)	0.0044 (8)	−0.0015 (7)
C26	0.0152 (9)	0.0126 (9)	0.0154 (10)	0.0006 (7)	0.0029 (8)	0.0007 (8)
C27	0.0265 (11)	0.0167 (10)	0.0151 (10)	0.0054 (9)	−0.0007 (9)	0.0057 (8)
C28	0.0377 (14)	0.0438 (15)	0.0351 (15)	0.0235 (12)	0.0108 (12)	0.0149 (12)
Cl1	0.01124 (19)	0.0134 (2)	0.0103 (2)	0.00477 (16)	0.00389 (16)	0.00282 (16)
Ir1	0.00779 (4)	0.00810 (4)	0.00644 (4)	0.00263 (3)	0.00083 (3)	0.00086 (3)
N1	0.0098 (7)	0.0095 (7)	0.0091 (7)	0.0035 (6)	0.0024 (6)	0.0017 (6)
N2	0.0107 (7)	0.0096 (7)	0.0065 (7)	0.0032 (6)	0.0008 (6)	0.0004 (6)
N3	0.0378 (11)	0.0250 (10)	0.0226 (10)	0.0156 (9)	0.0042 (9)	0.0067 (8)
O1	0.0454 (11)	0.0286 (9)	0.0291 (10)	0.0197 (8)	−0.0143 (8)	0.0008 (8)
O2	0.0343 (9)	0.0235 (8)	0.0257 (9)	0.0135 (7)	0.0150 (7)	0.0131 (7)
O3	0.0172 (7)	0.0168 (7)	0.0129 (7)	0.0100 (6)	−0.0048 (6)	−0.0009 (6)
S1	0.0108 (2)	0.0099 (2)	0.0079 (2)	0.00406 (16)	0.00035 (16)	0.00033 (16)

### Geometric parameters (Å, º)

**Table:**

C1—O1	1.211 (3)	C16—C17	1.416 (2)
C1—C2	1.477 (3)	C16—Ir1	2.0077 (18)
C2—C7	1.391 (3)	C17—C18	1.398 (2)
C2—C3	1.400 (3)	C17—C24	1.469 (2)
C3—C4	1.396 (3)	C18—C19	1.390 (3)
C4—C5	1.402 (3)	C20—N2	1.346 (2)
C4—Ir1	2.0466 (18)	C20—C21	1.388 (3)
C5—C6	1.397 (3)	C21—C22	1.391 (3)
C5—C12	1.470 (3)	C22—C23	1.383 (3)
C6—C7	1.392 (3)	C23—C24	1.394 (3)
C8—N1	1.351 (2)	C24—N2	1.366 (2)
C8—C9	1.376 (3)	C25—S1	1.7799 (19)
C9—C10	1.381 (3)	C26—S1	1.7870 (19)
C10—C11	1.384 (3)	C27—N3	1.141 (3)
C11—C12	1.390 (3)	C27—C28	1.453 (3)
C12—N1	1.366 (2)	Cl1—Ir1	2.4748 (5)
C13—O2	1.204 (2)	Ir1—N2	2.0609 (15)
C13—C14	1.481 (3)	Ir1—N1	2.0614 (15)
C14—C19	1.384 (3)	Ir1—S1	2.3810 (5)
C14—C15	1.402 (2)	O3—S1	1.4903 (13)
C15—C16	1.392 (2)		
			
O1—C1—C2	125.8 (2)	C20—C21—C22	119.06 (17)
C7—C2—C3	120.72 (18)	C23—C22—C21	119.21 (17)
C7—C2—C1	119.00 (18)	C22—C23—C24	119.65 (17)
C3—C2—C1	120.28 (18)	N2—C24—C23	120.64 (16)
C4—C3—C2	120.47 (18)	N2—C24—C17	113.70 (15)
C3—C4—C5	117.90 (17)	C23—C24—C17	125.66 (16)
C3—C4—Ir1	127.61 (14)	N3—C27—C28	179.7 (2)
C5—C4—Ir1	114.48 (13)	C16—Ir1—C4	89.71 (7)
C6—C5—C4	122.04 (17)	C16—Ir1—N2	80.48 (6)
C6—C5—C12	122.36 (17)	C4—Ir1—N2	89.53 (7)
C4—C5—C12	115.59 (16)	C16—Ir1—N1	93.83 (6)
C7—C6—C5	119.15 (18)	C4—Ir1—N1	79.63 (7)
C2—C7—C6	119.70 (18)	N2—Ir1—N1	167.83 (6)
N1—C8—C9	122.61 (18)	C16—Ir1—S1	90.41 (5)
C8—C9—C10	119.19 (19)	C4—Ir1—S1	179.68 (5)
C9—C10—C11	118.86 (18)	N2—Ir1—S1	90.19 (4)
C10—C11—C12	120.15 (18)	N1—Ir1—S1	100.65 (4)
N1—C12—C11	120.48 (17)	C16—Ir1—Cl1	177.65 (5)
N1—C12—C5	113.96 (16)	C4—Ir1—Cl1	91.58 (5)
C11—C12—C5	125.56 (17)	N2—Ir1—Cl1	97.56 (4)
O2—C13—C14	126.05 (18)	N1—Ir1—Cl1	88.33 (4)
C19—C14—C15	120.78 (17)	S1—Ir1—Cl1	88.295 (18)
C19—C14—C13	118.36 (17)	C8—N1—C12	118.67 (16)
C15—C14—C13	120.86 (17)	C8—N1—Ir1	124.98 (12)
C16—C15—C14	120.26 (17)	C12—N1—Ir1	116.32 (12)
C15—C16—C17	118.19 (16)	C20—N2—C24	119.49 (15)
C15—C16—Ir1	127.48 (13)	C20—N2—Ir1	124.76 (12)
C17—C16—Ir1	114.21 (13)	C24—N2—Ir1	114.59 (11)
C18—C17—C16	121.29 (17)	O3—S1—C25	106.03 (9)
C18—C17—C24	123.30 (17)	O3—S1—C26	107.19 (9)
C16—C17—C24	115.18 (15)	C25—S1—C26	98.72 (9)
C19—C18—C17	119.24 (17)	O3—S1—Ir1	119.58 (6)
C14—C19—C18	120.16 (17)	C25—S1—Ir1	111.64 (7)
N2—C20—C21	121.84 (17)	C26—S1—Ir1	111.52 (7)

### Hydrogen-bond geometry (Å, º)

**Table:**

*D*—H···*A*	*D*—H	H···*A*	*D*···*A*	*D*—H···*A*
C19—H19···O3^i^	0.93	2.48	3.179 (3)	132
C25—H25*B*···N3^ii^	0.96	2.53	3.403 (3)	150
C26—H26*A*···O3^iii^	0.96	2.48	3.406 (2)	161
C28—H28*C*···O1^iv^	0.96	2.54	3.490 (3)	173

Symmetry codes: (i) *x*−1, *y*, *z*; (ii) −*x*+1, −*y*+2, −*z*+1; (iii) −*x*+2, −*y*+2, −*z*+2; (iv) *x*+1, *y*, *z*.
